# Inhomogeneous Point-Processes to Instantaneously Assess Affective Haptic Perception through Heartbeat Dynamics Information

**DOI:** 10.1038/srep28567

**Published:** 2016-06-30

**Authors:** G. Valenza, A. Greco, L. Citi, M. Bianchi, R. Barbieri, E. P. Scilingo

**Affiliations:** 1 Neuroscience Statistics Research Laboratory, Massachusetts General Hospital, Harvard Medical School, Boston, MA, USA; 2 Department of Information Engineering and Research Centre E Piaggio, University of Pisa, Pisa, Italy; 3 School of Computer Science and Electronic Engineering, University of Essex, Colchester, CO43SQ, UK; 4 Department of Advanced Robotics, Istituto Italiano di Tecnologia, via Morego 30, 16163 Genova, Italy

## Abstract

This study proposes the application of a comprehensive signal processing framework, based on inhomogeneous point-process models of heartbeat dynamics, to instantaneously assess affective haptic perception using electrocardiogram-derived information exclusively. The framework relies on inverse-Gaussian point-processes with Laguerre expansion of the nonlinear Wiener-Volterra kernels, accounting for the long-term information given by the past heartbeat events. Up to cubic-order nonlinearities allow for an instantaneous estimation of the dynamic spectrum and bispectrum of the considered cardiovascular dynamics, as well as for instantaneous measures of complexity, through Lyapunov exponents and entropy. Short-term caress-like stimuli were administered for 4.3–25 seconds on the forearms of 32 healthy volunteers (16 females) through a wearable haptic device, by selectively superimposing two levels of force, 2 N and 6 N, and two levels of velocity, 9.4 mm/s and 65 mm/s. Results demonstrated that our instantaneous linear and nonlinear features were able to finely characterize the affective haptic perception, with a recognition accuracy of 69.79% along the force dimension, and 81.25% along the velocity dimension.

Emotions such as anger, fear, and love can be communicated through touch to the forearm^1^,^2^. Indeed, the human tactile system is very sensible to affective stimuli like caressing, stroking or patting, thanks to the activity of low-threshold mechanoreceptors called CT fibers^1^,^2^. Through this knowledge, the relationship between the physical characteristics of a haptic stimulus and its pleasantness was previously studied^3^,^4^,^5^. Most pleasant stimuli were associated to the lowest applied force^3^, with movements velocity in the range 1–10 cm/s (3 cm/s considered as the most pleasant)^4^. Of note, these findings are consistent with stimuli administered in different parts of the body (forehead, arm, palm, thigh)^6^.

The dynamics of hearth contractions, which is modulated by the Autonomic Nervous System (ANS) activity, is significantly affected by emotion elicitation and by passive touch^7^,^8^,^9^,^10^,^11^,^12^,^13^,^14^,^15^. Specifically, linear and nonlinear analysis of Heart Rate Variability (HRV)^16^ revealed parasympatetic changes after massage^13^,^17^,^18^. Of note, the use of nonlinear quantifiers is justified by the fact that heartbeat dynamics shows exemplary nonlinear behavior, mainly generated through integration of multiple neural signaling at the level of the sinoatrial node^19^.

Major shortcomings of current signal processing methodologies used to objectively assess affective haptic stimuli are related to the stimulus duration. Standard linear and nonlinear HRV measures, in fact, require relatively long-term recordings to accurately characterize the emotional state of a subject. For example, observations from 30 seconds to 5 minutes are the least needed for simple HRV spectral analysis^16^. Nevertheless, actual affective haptic stimuli, such as caresses, usually last for few seconds. Therefore, standard signal processing methodologies are not suitable to perform such an assessment. Even considering affective haptic stimuli lasting for more than 30 seconds, the actual emotional perception will be compromised by the saturation of the CT fibers activity^3^,^5^. Furthermore, from a methodological point of view, standard methods are generally not suitable to provide accurate nonlinear fitting in the absence of information regarding the system phase space, and their estimates are biased by noise statistics (e.g., white or 1/*f* noise) underlying physiological dynamics, and interpolation techniques^20^,^21^.

To overcome these limitations, we here propose the application of inhomogeneous point-process models of heartbeat dynamics to assess short-term affective haptic perception in an instantaneous fashion, such that caress-like stimuli lasting for 4.3–25 seconds can be therefore automatically recognized. As an input, the proposed methodology takes unevenly sampled heartbeat interval information exclusively, derived from the electrocardiogram. To our knowledge, the use instantaneous measures of linear and nonlinear autonomic control derived from cardiovascular dynamics in order to characterize and automatically discern physical properties of affective haptic perception is novel in the current literature. Motivations of this study are related to scientific knowledge, and possible potential application in the field of experimental psychology and assessment of patients with mental disorders.

The model underlying these estimates comprises probability density functions (pdf) characterizing and predicting the time until the next event occurs, as a function of the past history. Of note, the derived instantaneous measures can be estimated without applying any interpolation techniques to the original series, and are associated to effective goodness-of-fit measures^20^,^21^,^22^,^23^. The framework relies on inverse-Gaussian point-processes with Laguerre expansion of the nonlinear Wiener-Volterra kernels, accounting for the long-term information given by the past heartbeat events^20^,^21^,^23^. This methodology has been successfully applied to the study of brain-heart interaction^24^, assessment of Parkinson’s disease^25^ and depression in bipolar disorder^26^,^27^.

In this study, the use of instantaneous nonlinear estimates is emphasized. We consider up to cubic-order nonlinearities, allowing for a comprehensive assessment through the dynamic spectrum and bispectrum of the considered cardiovascular dynamics^23^, as well as for instantaneous measures of complexity, through Lyapunov exponents and entropy^20^,^21^. As mentioned above, although the underlying physiological dynamics linked to these measures is still unknown, it has been widely accepted that the quantification of ANS nonlinear and complex dynamics provides meaningful information on psychophysiological and pathological states^28^,^29^,^30^,^31^,^32^. Furthermore, measures of time-varying complexity have enhanced discriminant power with respect to standard complexity measures^20^,^21^.

Short-term caress-like stimuli were administered on the forearms of 32 healthy volunteers (16 females) through an ad-hoc wearable haptic device able to mimic caresses^33^. In order to develop a processing chain able to actually discern affective perception using electrocardiogram-derived information exclusively, we moved from looking for simple statistical differences between stimulus physical characteristics to building an automatic classification algorithm.

Next, we describe the mathematical bases of the inhomogeneous point-process modeling, extending the previously proposed approach reported in^23^. Then, a brief description of related instantaneous estimates is reported, allowing with experimental set-up and results which are expressed in terms of confusion matrix of classification and complementary statistics.

## Materials and Methods

### Point-Process Model of the Heartbeat

A random point process is a stochastic process characterizing the occurrence in time of discrete events. Here, this methodology aims to deriving estimates from the intrinsically discrete, unevenly sampled heartbeat events at each moment in time. To this extent, starting from the electrocardiogram (ECG), it is possible to define the pdf predicting the next ventricular contraction (R-peaks), through a parametric formulation of the past heartbeat events. As such a pdf is a continuous function defined in the time domain, it is possible to obtain instantaneous cardiovascular estimates at any desired time resolution.

Mathematically, motivated by both goodness-of-fit and physiological reasons, the pdf characterizing cardiovascular control dynamics follows an inverse-Gaussian model^20^,^21^,^23^:


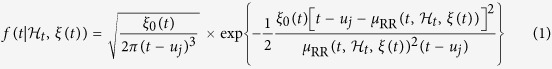


with:

• *t* ∈ (0, *T*], the observation interval;

• 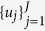 the R-wave events, in this study, detected from the ECG, and 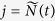 the index of the previous R-wave event before time *t*

• 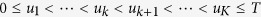 the times of the events;

• RR_*j*_ = *u*_*j*_ − *u*_*j*−1_ > 0 the *j*^*th*^ RR interval;

• *N*(*t*) = max{*k* : *u*_*k*_ ≤ *t*} the sample path of the counting process of the RR interval seris;

• 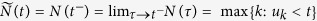


• 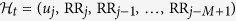;

• *ξ*(*t*) the vector of the model time-varying parameters;

• 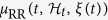 the first-moment statistic (mean) of the distribution;

• *ξ*_0_(*t*) > 0 the shape parameter of the inverse Gaussian distribution;

Accounting for history dependence, 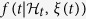 is thus able to predict the next heartbeat event, being parametrized in its first-order moment as a Nonlinear Autoregressive model with Laguerre expansions (NARL) of the Volterra terms:


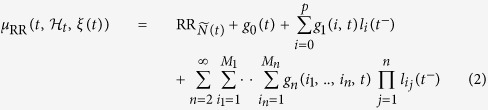


where


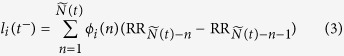


is the output of the Laguerre filters just before time *t*, and


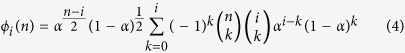


is the *i*^th^-order discrete time orthonormal Laguerre function, with *n* ≥ 0 and *α* is the discrete-time Laguerre parameter (0 < *α* < 1) which determines the rate of exponential asymptotic decline of these functions. The coefficients *g*_0_, {*g*_1_(*i*)}, and {*g*_2_(*i*, *j*)} correspond to the time-varying zero-, first-, second-order NARL coefficients, respectively. Considering the derivative R-R series improves the achievement of stationarity within the sliding time window *W* (in this work we have chosen *W* = 90 sec.)^23^.

Of note, the corresponding nonlinear autoregressive (NAR) Wiener-Volterra model with degree of nonlinearity 2 and long-term memory^34^ becomes:


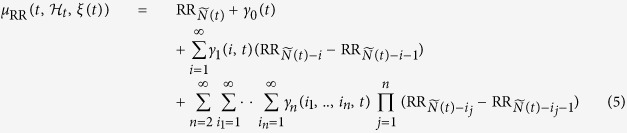


As mentioned above, as 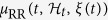 is defined in continuous time, it is possible to obtain an instantaneous R–R mean estimate at a very fine timescale, which requires no interpolation between the arrival times of two beats. In this work, we consider nonlinearities associated to eq. 5 up to the third-order. Cubic terms, in fact, allow for the estimation of the dominant Lyapunov exponent, whereas quadratic terms account for the high-order spectral estimation (see sections below).

#### Parameter Estimation, Model Selection, Goodness-of-Fit

The optimal time-varying parameter vector ***ξ***(*t*) is defined as the set maximizing the following local log-likelihood^23^:


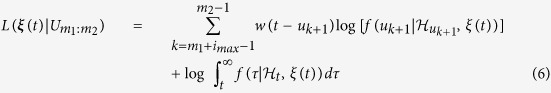


where:

• (*t* − *l*, *t*] is a local observation interval of duration *l*;

• 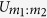 is a subset of R-wave events;

• *m*_1_ = *N*(*t* − *l*) + 1;

• *m*_2_ = *N*(*t*);

• *i*_*max*_ = *max*{*i*_1_, *i*_2_, …, *i*_*n*_};

• *w*(*τ*) = *e*^−*ϖτ*^ is an exponential weighting function.

We use a Newton-Raphson procedure to maximize the local log-likelihood in eq. 6. This formulation is also used to preprocess all the actual heartbeat data with a previously developed algorithm^35^, performing a real-time R-R interval error (e.g., peak detection errors and ectopic beats) detection and correction.

Because there is significant overlap between adjacent local likelihood intervals, we start the Newton-Raphson procedure at *t* with the previous local maximum-likelihood estimate at time *t* − Δ, where Δ defines the time interval shift to compute the next parameter update.

We determine the optimal model orders based on the Kolmogorov-Smirnov (KS) test and associated KS statistics^23^. Autocorrelation plots are also considered to test the independence of the model-transformed intervals^23^.

### Instantaneous Time, Frequency, and Higher-Order Spectral Analysis

#### Estimation of the Input-Output Volterra Kernels

In order to provide quantitative tools related to representations defined in the time, frequency, and higher order spectral domains, considering quadratic nonlinearities (with n = 2), estimated parameters of the fully autoregressive eq. 5 have to be linked to classical input-output Wiener-Volterra model^23^.

This transformation can be performed in the frequency domain by using the following relationships^23^


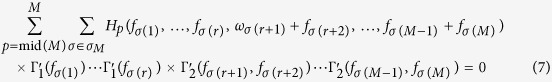


where

• *H*_*p*_(*f*_1_, …, *f*_*n*_) is the Fourier transforms of the Wiener-Volterra kernels of order *p*

• 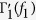 and 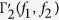 are the Fourier transforms of the extended terms of *γ*_1_(*i*) and *γ*_2_(*i*, *j*)^23^, respectively

• *M* is a given integer representing the kernel order

• 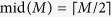


• *r* = 2*p* − *M* and *σ*_*M*_ is the permutation set of 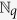


Since the Volterra kernels induced by the NAR model are nested, the *M*^th^-order kernel can be deduced recursively^23^.

#### Instantaneous Analysis in the Time and Frequency Domain

The time-domain characterization is based on the first and the second order moments of the underlying probability structure^22^. Namely, given the time-varying parameter set ***ξ***(*t*), the instantaneous estimates of mean 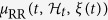, R-R interval standard deviation 
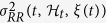
, mean heart rate 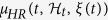, and heart rate standard deviation 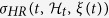 can be derived at each moment in time as follows^22^:


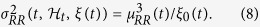



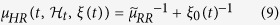



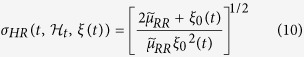


The linear power spectrum can be estimated as:


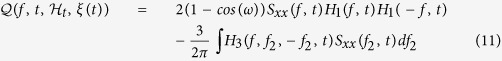


where 
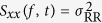
.

By integrating the (11) in each frequency band, we can compute the index within the very low frequency (VLF = 0.01–0.05 Hz), low frequency (LF = 0.05–0.15 Hz), and high frequency (HF = 0.15–0.5 Hz) ranges^22^.

#### Instantaneous Bispectral Analysis

The higher-order spectral representation allows for statistics beyond the second order, and phase relations between frequency components otherwise suppressed.

The analytical solution for the bispectrum of a nonlinear system response subject to stationary, zero-mean Gaussian input can be found in^36^.

Further details on the instantaneous bispectrum derivation from point-process nonlinear models can be found in^23^.

Through this powerful computational tool, we here evaluate the instantaneous presence of nonlinearity in heartbeat series by calculating the nonlinerar sympatho-vagal interactions. Specifically, by integrating |Bis(*f*_1_, *f*_2_, *t*)| in the appropriate frequency bands, it is possible to obtain:


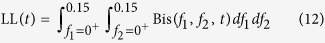



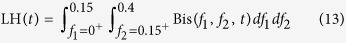



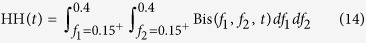


### Instantaneous Measures of Complexity

#### Instantaneous Lyapunov Exponents

Considering cubic nonlinearities (with n = 3) in the fully autoregressive eq. 5, and using a Fast Orthogonal Search algorithm, it is possible to estimate the complete Lyapunov Exponents (LE) spectrum at each moment in time^21^. In this study, we use the Instantaneous Dominant Lyapunov Exponent (IDLE, *λ*_1_), which is the first exponent of the LE spectrum, along with *λ*_2_:


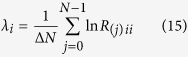


where Δ is the sampling time step, *N* the length of data samples, and *R*_(*j*)*ii*_ is part of the QR decomposition of the Jacobian of the time series^21^.

#### Instantaneous Entropy Measures

In this study, the estimated instantaneous entropy measures refer to the inhomogeneous point-process approximate and sample entropy, *A*_I_ and *S*_I_, respectively^20^. These measures have their foundation in the instantaneous phase space estimation, in which the distance between two points is calculated through KS distance (i.e. the maximum value of the absolute difference between two cumulative distribution functions) between the two pdfs associated to these points. The time-varying radius *r*(*t*) is instantaneously expressed as 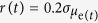
20.

Moreover, *A*_I_ and *S*_I_ dynamical values are not seriously affected by the kind of noise underlying the complex system, thus ensuring truly instantaneous tracking of the dynamic system complexity^20^.

### Experimental Setup

Thirty-two participants aged 27 ± 2 (16 females) gave their informed consent to take part in the study. No participants reported physical limitations and any experience of mental or personality disorder in their life that would affect the experimental outcomes. All participants gave written informed consent to participating in the study, which was approved by the Ethical Committee of the University of Pisa-Pisa University Hospital, Pisa (Italy). All experimental procedures and analyses were carried out in accordance with such approved guidelines. During the experimental protocol the participants were comfortably seated, the right forearm was horizontal and placed on the forearm support, hand palm down. For all trials, participants wore earplugs in order to prevent any auditory cues.

**Figure 1:**
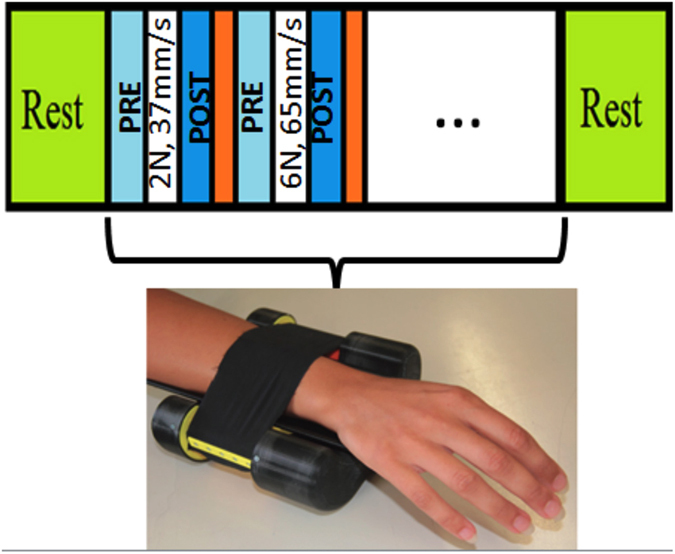
(Top) Experimental protocol timeline and (bottom) the haptic system worn by a subject.

The ad-hoc robotic device uses a layer of elastic fabric to convey haptic like-caressing stimuli^33^ (see Fig. 1). The system is endowed with a load cell placed at the basis of the forearm support, prior to the experiment, the load cell was auto calibrated with respect to the forearm weight. When the device was active, two different operating phases were distinguishable: a calibration phase, when forces exerted by the fabric on the user were calibrated, and a movement phase. In this phase, the motors started to coherently rotate and the fabric moves forward and backward over the user forearm, simulating a caress. For further technical details on the device, the reader is invited to refer to ref. 33.

In this study, we considered 4 different stimuli among 2 levels of force (F1 = 2 N, F2 = 6 N) and 2 levels of velocity (V1 = 9.4 mm/s, V2 = 65 mm/s), obtained by feeding the motors with two different sinusoidal input trajectories, at the frequencies of 0.1 Hz and 0.4 Hz. These values were chosen according to previous studies^4^,^33^. Exemplary timeline is shown in Fig. 1. Between two stimuli, the motors were stopped and the force was set to 0 N, in this case the fabric was only lightly in contact with the forearm. As the force increases, the fabric is more closely wrapped around the forearm and there is no more pure sliding (as with light forces) but also skin torsion. This behavior was coherent with the goal to reproduce as exhaustively as possible the behavior of the human caress. Throughout the experiment, there were two phases of resting sessions with a duration of two minutes: the first at the beginning of the protocol, the second at the end of the stimulation.

The four kinds of stimulation were suitably randomized, with a pre-stimulus and a post-stimulus interval of 35 seconds each. During the elicitation, the ECG was continuously acquired, following the Einthoven triangle configuration, by means of a dedicate hardware module, i.e. the ECG100C Electrocardiogram Amplifier from BIOPAC inc. with a sampling rate of 500 Hz. To obtain the HRV series from the ECG, a QRS complex detection algorithm was used, i.e. the automatic algorithm developed by Pan-Tompkins^37^. Peak detection and consequent correction of ectopic beats was performed with an automatic technique previously described in ref. 35.

#### Statistical Analysis and Pattern Recognition

All features were instantaneously calculated with a Δ = 5 ms temporal resolution. We considered the time-varying dynamics of a given feature *X* estimated throughout the short-time window when the affective stimulus occurs. Although the length of such a short-time window depends on the caressing velocity ranging from 4.3 to 25 seconds, in order to avoid possible biases related to signal length, we considered feature values derived from the last second of each caressing stimulus. In order to reduce possible intra-, and inter-subject variability, this value was then normalized by the feature value gathered from the fist 10 s of the initial resting state. Quantification of the feature dynamical information was performed through the maximum value 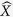, the median 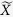 and its respective absolute deviation 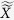, all calculated along the time of last second of caressing. Accordingly, since we derived 13 instantaneous features, the total number of parameter used for the force/velocity classification was 39. A summary of all considered features is reported in Table 1. The median 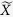 is considered as a measure of central tendency, which can be related to classical estimation methods that provide one finite value when considering a given time window, whereas the median absolute deviation 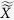 and the maximum value 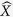 depend on the feature variability along the time. Then, to average among multiple subjects, we consider group values expressed as median and its respective absolute deviation (i.e., for a feature 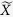, 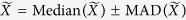 where 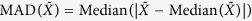).

As an exploratory preliminary step, for each feature, we evaluated the statistical differences between groups of caresses, identified by levels of force/velocity. The difference was expressed in terms of p-values from a non-parametric Wilcoxon test for paired data, under the null hypothesis that the medians of the two sample groups are equal.

**Figure 2:**
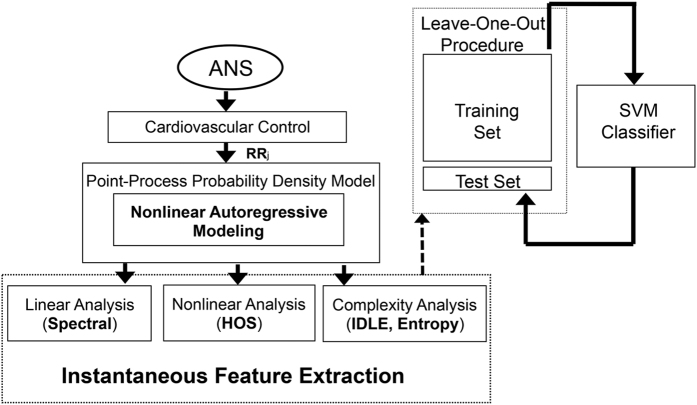
Overview of the signal processing and classification chain. ANS dynamics on cardiovascular control modulates the heartbeat dynamics. Starting from data acquisition, RR series are extracted by using automatic peak detection algorithms applied on artifact-free signals. The nonlinear point-process model is fitted to the HRV series, and all features are estimated in an instantaneous fashion. Successively, for each subject, a feature set is defined and fed into support vector machine-based classification using leave-one our procedures.

In order to actually discern between force and velocity of the caresses using heartbeat dynamics exclusively, an automatic classification algorithm is needed. To this extent, each feature constituted a single dimension of the feature space. A multidimensional point was considered an outlier if z-scores associated to its dimensions were greater than 4. The obtained feature set is taken as an input of the Leave-One-Out (LOO) procedure applied on a Support Vector Machine (SVM)-based pattern recognition^38^ (see Fig. 2). Within the LOO scheme, the training set was normalized by subtracting the median value and dividing by the MAD over each dimension. More specifically, we used a nu-SVM (nu = 0.5) having a radial basis kernel function with *γ* = *n*^−1^, with n = 39 equal to the number of features.

Additionally, in order to explore the relative importance of all features in the classification problem, we employed a support vector machine recursive feature elimination (SVM-RFE) procedure in a wrapper approach (RFE was performed on the training set of each fold and we computed the median rank for each feature over all folds). We specifically chose a recently developed nonlinear SVM-RFE which employes a radial basis function kernel and includes a correlation bias reduction strategy into the feature elimination procedure^39^.

Classification results are here expressed in terms of recognition accuracy, and in form of confusion matrix. The generic element *r*_*ij*_ of the confusion matrix indicated a percentage as to how many times a pattern belonging to class *i* was classified as belonging to class *j*. A more diagonal confusion matrix corresponded to a higher degree of classification. All of the algorithms were implemented by using *Matlab*© v8.4 endowed with an additional toolbox for pattern recognition, i.e., LIBSVM^40^.

In the next section, we report experimental results in classifying force and velocity level of the administered caress-like stimuli. Specifically, we show best classification results given the ad-hoc number of feature identified through the SVM-RFE procedure.

## Results

**Figure 3:**
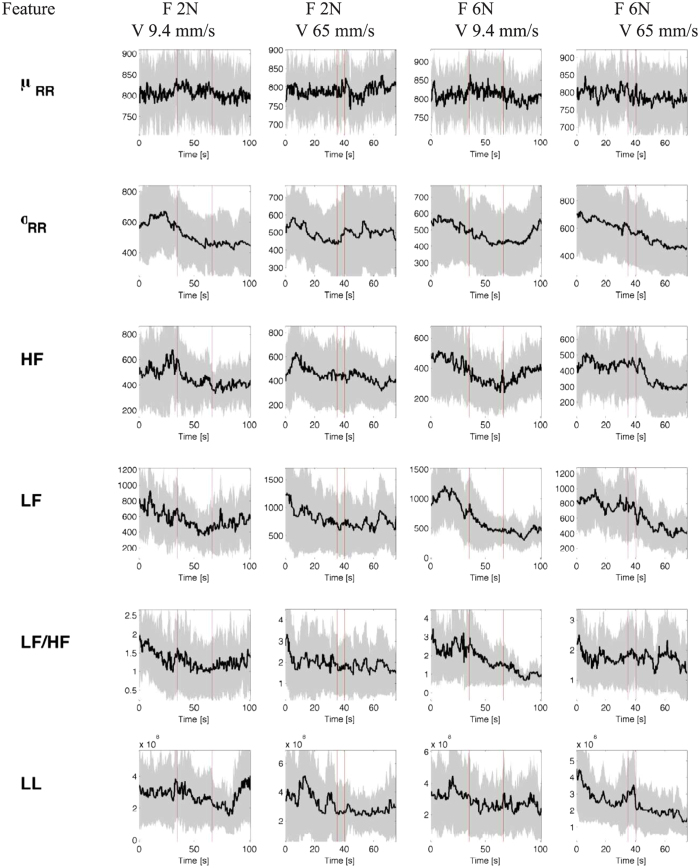
Instantaneous heartbeat estimates computed from a representative subject, obtained through a point-process NARL model. Black lines and gray areas indicate median and MAD values among subjects. Vertical red lines mark the short-time window of caressing. From the top panel, the estimated mean, *μ*_RR_(*t*), the RR standard deviation, *σ*_RR_(*t*), the high frequency (HF), the low frequency (LF), the (LF/HF) ratio, and LL are reported.

**Figure 4:**
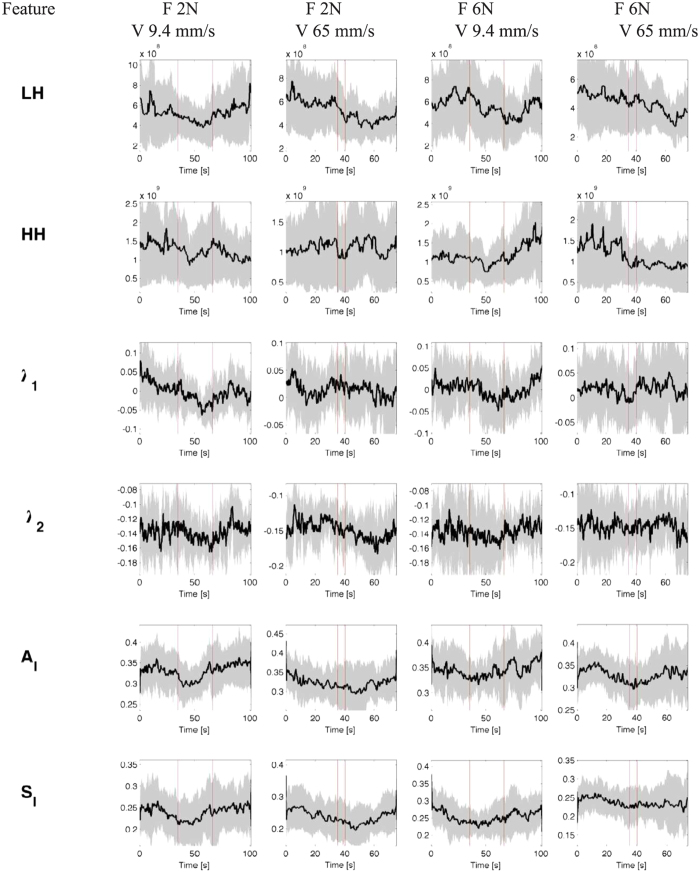
Instantaneous heartbeat estimates computed from a representative subject, obtained through a point-process NARL model. Black lines and gray areas indicate median and MAD values among subjects. Vertical red lines mark the short-time window of caressing. From the top panel, LL and HH bispectral statistics, the instantaneous first and second Lyapunov exponent, *λ*_1_ and *λ*_2_, respectively, and the instantaneous point-process entropy measures *A*_*I*_ and *S*_*I*_ are reported.

**Figure 5:**
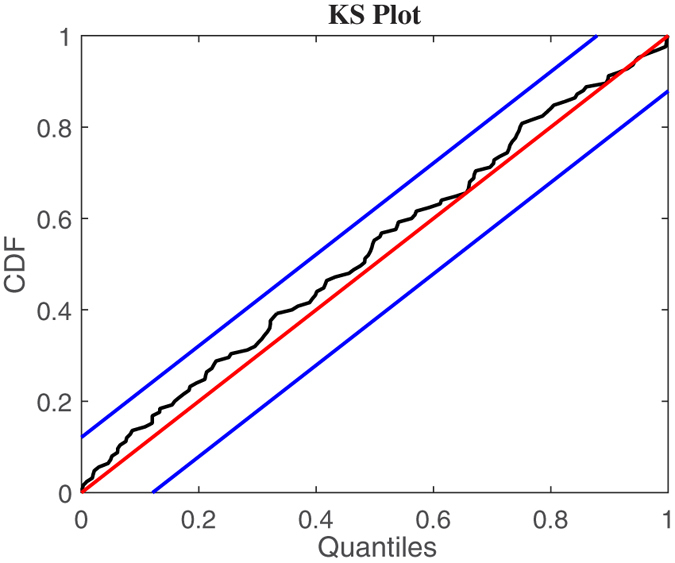
KS plot using the proposed inhomogeneous point process nonlinear models for a representative subject (N. 1) undergoing caressing-like stimuli. Blue diagonal and red lines indicate the 95% confidence bounds.

**Figure 6:**
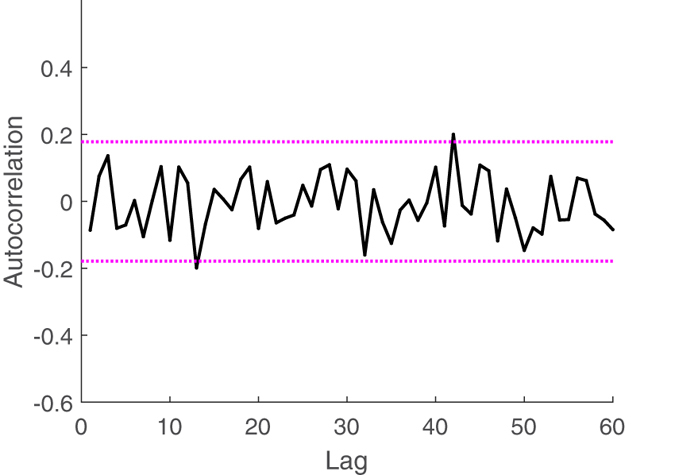
Autocorrelation plot using the proposed inhomogeneous point process nonlinear models for a representative subject (N. 1) undergoing caressing-like stimuli. Dashed lines indicate the 95% confidence bounds.

Instantaneous series from a representative subject are shown in Figs 3 and 4. The model order selection analysis revealed optimal NARL order as *p* = 3 and *M*_1_ = 1, *M*_2_ = 0. Over all the considered subjects, 31 out of a total of 32 recordings showed KS plots and more than 98% of the autocorrelation samples within the 95% of the confidence interval. Of note, KS distances were as low as 0.0313 ± 0.0062. Figures 5 and 6 show an exemplary Kolmogorow-Smirnov and autocorrelation plots demonstrating how our model well predicted all heartbeat events of a given RR interval series.

As a first preliminary investigation, we performed a statistical analysis of all the features between force and velocity levels. Significant results (*p* < 0.05) from such an analysis are shown in Table 2.

Significant differences were found in discerning caresses performed at different velocities only. No relevant changes, in fact, were found in instantaneous heartbeat linear and nonlinear dynamics between caresses performed at different force levels. On the other hand, caresses performed at higher velocity affected the variability of all of the instantaneous measures, as well as median value of four features, one of which was related to heartbeat complex dynamics, and maximum value of 8 features, six of which were related to heartbeat complex/nonlinear dynamics (see Table 2).

### Caressing Force Classification

Table 3 shows the confusion matrix as well as the total average accuracy whilst discerning caresses-like stimuli performed at different *force* levels, considering first 26 features selected by the nonlinear SVM-RFE algorithm. This specific feature set, giving the best total average accuracy, which was 69.79%, is partially listed in Table 4 ordered by median rank over every fold computed during the LOO procedure.

**Figure 7:**
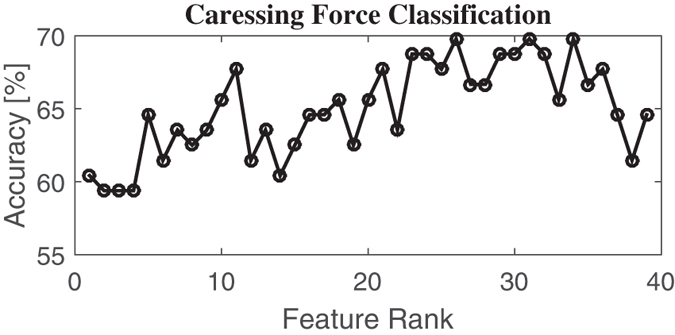
Recognition accuracy in discerning caressing force levels as a function of the feature rank estimated through the SVM-RFE procedure.

Figure 7 shows the recognition accuracy while discerning caressing force levels, as a function of the feature rank estimated through the SVM-RFE procedure.

Furthermore, we performed the classification of caressing at low force vs. high force levels using features only related to the central tendency (median) of the time-varying estimates, such as 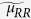, 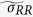, 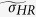, 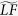, 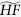, and 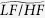, 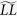, 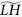, 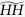, 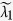, 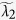, 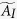, 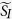, avoiding any measure of variability (i.e., MAD and maximum values). Results of this classification are shown in Table 5. In this case, the total average accuracy was 57.66%.

### Caressing Velocity Classification

Table 6 shows the confusion matrix as well as the total average accuracy whilst discerning caresses performed at different *velocity* levels, considering first 35 features selected by the nonlinear SVM-RFE algorithm. This specific feature set, giving the best total average accuracy, which was 81.25%, is partially listed in Table 7 ordered by median rank over every fold computed during the LOO procedure.

**Figure 8:**
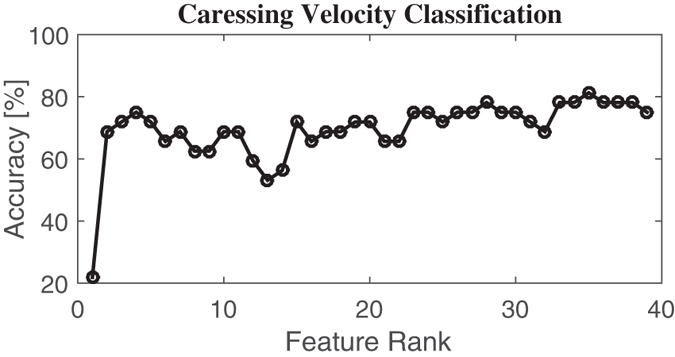
Recognition accuracy in discerning caressing velocity levels as a function of the feature rank estimated through the SVM-RFE procedure.

Figure 8 shows the recognition accuracy, while discerning caressing velocity levels, as a function of the feature rank estimated through the SVM-RFE procedure.

Likewise performed for the caressing force classification, we performed the classification of caressing at low velocity vs. high velocity levels using features only related to the central tendency (median) of the time-varying estimates, avoiding any measure of variability. Results of this classification are shown in Table 8. In this case, the total average accuracy was 57.14%.

## Discussion and Conclusion

In conclusion, we proposed a comprehensive signal processing framework to assess short-term affective haptic stimuli through the analysis of heartbeat dynamics exclusively. We built on a previously proposed set of equations defining inhomogeneous point-process nonlinear models^23^ to obtain instantaneous linear, nonlinear, and complex estimates of cardiovascular dynamics. Key concepts relay on the definition of a continuous pdf predicting the next heartbeat event, identified through R-waves from the ECG, parametrized using a nonlinear autoregressive model with Laguerre expansion of the Wiener-Volterra terms^23^. Such a parametrization is defined up to the cubic terms, allowing for the instantaneous estimation of Lyapunov exponents^21^ and approximate and sample entropy^20^, as well as instantaneous nonlinear bispectral measures, and linear estimates defined in the time and frequency domain^23^.

To our knowledge, this is the only signal processing method proposed so far able to objectively assess short-term (4.3 to 25 seconds) affective haptic stimuli such as caresses. Standard linear and nonlinear HRV measures^16^, in fact, would be unable to perform such an assessment as they require relatively long-term recordings to accurately characterize the emotional state of a subject. On the other hand, recognition of long-term caressing (i.e., >30 seconds) would lead to the saturation of the CT fibers activity^3^,^5^, strongly biasing the affective part of the emotional perception. Of note, motivation of this study are strictly related to objective assessments performed in experimental psychology and, more in general, in the evaluation of patients with mental disorders.

Our analysis revealed that instantaneous cardiovascular dynamics is significantly affected by the velocity of the affective haptic stimuli (see Table 2). As a matter of fact, no relevant changes were found between caresses performed at different force levels. Of note, 14 out of the 25 features which were sensitive to caressing velocity level are derived in a nonlinear fashion, revealing a decrease of complexity variability and maximum values during the haptic stimulus. This result further justify the use of real-time estimates of heartbeat dynamics through point-process modeling, also highlighting the great role of nonlinear dynamics in cardiovascular control, fervently pointed out in the current literature^16^. In order to develop a processing chain able to actually discern affective perception, we moved from looking for simple statistical differences between stimulus physical characteristics to building an automatic classification algorithm. The automatic classification, embedded in the on-line processing chain, was performed by means of nu-SVM, and following a LOO and nonlinear SVM-RFE procedures for cross-validation and feature selection, respectively. Recognition accuracies were very satisfactory, being of 69.79% along the force dimension, and 81.25% along the velocity dimension. This suggest that, in line with results from the statistical analysis, cardiovascular dynamics is strongly affected by the caressing velocity, rather than the caressing force. In order to further emphasize the role of our processing framework, which allows for the calculation of features’ variability measures through the definition of instantaneous estimates, we performed the caressing force and velocity classification using features only related to the central tendency. In these cases, recognition accuracy were as low as <58%. This result demonstrates that information on central tendency only are not sufficient to achieve an automatic discrimination of affective haptic perception using heartbeat dynamics exclusively. This is also demonstrated by the results of the SVM-RFE feature selection procedure, pointing out how complexity variability measures are the most needed for the classification (see Tables 4 and 7). Indeed, as shown in Fig. 3, the time-varying dynamics of the heartbeat features is highly non-stationary, with most significant changes in nonlinear and complex dynamics (i.e., LL, LH, HH, *λ*_1_, *λ*_2_, *A*_*I*_, *S*_*I*_) during caressing at slower velocity. Importantly, our results are not biased by possible stochastic components underlying physiological dynamics, as our instantaneous complexity measures are not significantly affected by the statistical properties of the physiological noise behind the observed dynamics^20^,^21^.

Finally, we remark that in this study caressing-like stimuli were administered through an ad-hoc robotic device^29^. Further research is needed to investigate possible differences/similarities with actual caresses by a human hand, possibly investigating more than two levels of caressing force and velocity.

## Additional Information

**How to cite this article**: Valenza, G. *et al*. Inhomogeneous Point-Processes to Instantaneously Assess Affective Haptic Perception through Heartbeat Dynamics Information. *Sci. Rep.* **6**, 28567; doi: 10.1038/srep28567 (2016).

## Figures and Tables

**Table 1:** A summary of all features used in this study.

Feature Symbol	Description	Meaning	Reference
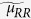 , 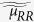 , 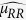	Mean of the Inverse-Gaussian pdf	Instantaneous Mean of the RR Interval Series	22,23
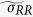 , 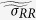 , 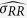	Standard Deviation of the Inverse-Gaussian pdf	Instantaneous Standard Deviation of the RR Interval Series	22,23
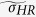 , 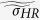 , 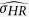	Heart rate Standard Deviation	Instantaneous Std of the Heartbeat Series	22,23
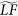 , 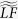 , 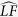	Low-Frequency Power of the RR interval series spectrum	Instantaneous Sympathetic and Parasympathetic Activity	22,23
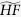 , 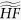 , 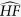	High-Frequency Power of the RR interval series spectrum	Instantaneous Parasympathetic Activity	22,23
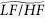 , 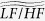 , 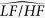	Ratio between Low- and High-Frequency Power of the RR interval series spectrum	Instantaneous Sympatho-Vagal Balance	22,23
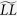 , 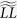 , 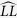	Low-Freq./Low-Freq. integration of the RR interval series bispectrum	Instantaneous Nonlinear Sympatho-Vagal Interactions	23
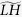 , 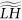 , 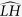	Low-Freq./High-Freq. integration of the RR interval series bispectrum	Instantaneous Nonlinear Sympatho Vagal Interactions	23
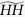 , 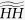 , 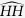	High-Freq./High-Freq. integration of the RR interval series bispectrum	Instantaneous Nonlinear Sympatho-Vagal Interactions	23
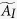 , 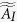 , 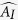	Inhomogeneous Point-Process Approximate Entropy of the RR interval series	Measure of Instantaneous Complexity	20
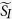 , 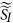 , 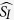	Inhomogeneous Point-Process Sample Entropy of the RR interval series	Measure of Instantaneous Complexity	20
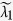 , 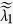 , 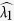	First Lyapunov Exponent of the RR interval series	Measure of Instantaneous Complexity	21
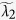 ,.., 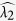	Second Lyapunov Exponent of the RR interval series	Measure of Instantaneous Complexity	21

**Table 2:** Significant features and p-values.

Feature	Velocity 9.4 mm/s	Velocity 65 mm/s	p-values
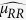	0.88 ± 0.11	0.84 ± 0.11	0.0075
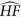	591.10 ± 357.82	547.99 ± 321.54	0.046
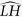	8.43 10^8^ ± 4.22 10^8^	5.09 10^8^ ± 2.5 10^8^	0.040
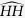	1.6 10^9^ ± 9.37 10^8^	1.29 10^9^ ± 8.3 10^8^	0.0076
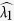	0.080 ± 0.074	0.056 ± 0.066	0.047
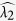	−0.064 ± 0.041	−0.11 ± 0.035	<10^−6^
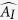	0.39 ± 0.051	0.33 ± 0.051	<7 * 10^−5^
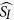	0.30 ± 0.059	0.24 ± 0.064	<4 * 10^−5^
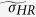	4.48 ± 1.89	5.47 ± 1.99	0.049
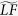	538.25 ± 354.08	706.40 ± 496.25	0.015
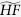	384.12 ± 231.03	451.63 ± 269.10	0.013
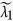	−0.0059 ± 0.057	0.016 ± 0.058	0.01
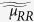	0.020 ± 0.0065	0.015 ± 0.0061	0.016
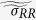	29.83 ± 18.92	9.24 ± 5.57	<10^−6^
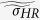	0.341 ± 0.17	0.138 ± 0.085	<10^−4^
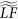	79.17 ± 48.63	29.71 ± 26.19	<10^−3^
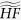	41.44 ± 31.21	16.21 ± 11.30	<10^−3^
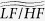	0.30 ± 0.23	0.12 ± 0.089	<10^−3^
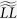	5.08 10^7^ ± 3.8 10^7^	2.27 10^7^ ± 2.02 10^7^	0.041
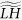	6.88 10^7^ ± 3.76 10^7^	2.29 10^7^ ± 1.09 10^7^	<10^−4^
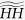	1.18 10^8^ ± 8.05 10^7^	5.52 10^7^ ± 4.57 10^7^	<10^−5^
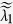	0.026 ± 0.0079	0.011 ± 0.0044	<10^−6^
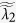	0.020 ± 0.0035	0.011 ± 0.0040	<10^−6^
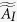	0.021 ± 0.0077	0.0060 ± 0.0034	<10^−6^
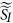	0.019 ± 0.0080	0.0057 ± 0.0033	<10^−6^

p-values are gathered from the Wilcoxon non-parametric test.

**Table 3:** Confusion matrix for caressing administered at different *force* levels.

SVM	Force 2 N	Force 6 N
**Force 2 N**	76.09	23.91
**Force 6 N**	36.00	64.00

Values are expressed as percentages. Total Accuracy: 69.79%.

**Table 4:** Selected features ordered by their median rank over every fold computed during the LOO procedure for *force* classification.

Rank	Feature
1	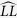
2	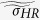
3	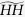
4	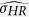
5	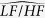
6	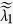

**Table 5:** Confusion matrix for caressing at different *force* levels, without features related to instantaneous variability.

SVM	Force 2 N	Force 6 N
**Force 2 N**	53.70	46.30
**Force 6 N**	38.60	61.40

Values are expressed as percentages. Total Accuracy: 57.66%.

**Table 6:** Confusion matrix for caressing administered at different *velocity* levels.

SVM	Velocity 9.4 mm/s	Velocity 65 mm/s
**Velocity 9.4 mm/s**	86.67	13.33
**Velocity 65 mm/s**	23.53	76.47

Values are expressed as percentages. Total Accuracy: 81.25%.

**Table 7:** Selected features ordered by their median rank over every fold computed during the LOSO procedure for *velocity* classification.

Rank	Feature
1	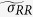
2	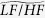
3	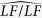
4	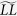
5	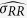
6	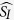

**Table 8:** Confusion matrix for caressing administered at different *velocity* levels, without features related to instantaneous variability.

SVM	Velocity 9.4 mm/s	Velocity 65 mm/s
**Velocity 9.4 mm/s**	58.33	41.67
**Velocity 65 mm/s**	43.90	56.10

Values are expressed as percentages. Total Accuracy: 57.14%.
